# An Unusual Occurrence of Synchronous Squamous Cell Carcinoma and Invasive Ductal Carcinoma in the Ipsilateral Breast: A Case Report

**DOI:** 10.2174/0115734056373786250527105407

**Published:** 2025-06-03

**Authors:** Seoyun Choi, Eun Jung Choi, Bo Ram Kim, Kyoung Min Kim

**Affiliations:** 1 Department of Radiology and Research Institute of Clinical Medicine of Jeonbuk National University-Biomedical Research Institute of Jeonbuk National University Hospital, Jeonbuk National University Medical School, Jeonju, Korea; 2 Department of Pathology, Jeonbuk National University Medical School, Research Institute of Clinical Medicine of Jeonbuk National University, Biomedical Research Institute of Jeonbuk National University Hospital, and Research Institute for Endocrine Sciences, Jeonju, Korea

**Keywords:** Breast neoplasms, Carcinoma, Squamous cell, Neoplasms, Multiple primary, Diagnostic imaging

## Abstract

**Background:**

The synchronous occurrence of primary pure squamous cell carcinoma (SCC) and invasive ductal carcinoma (IDC) of the breast is rare. Accurate identification of synchronous primary malignancies is crucial because their prognosis and treatment differ significantly from recurrent diseases. Herein, we present an unusual case highlighting the synchronous development of primary SCC and IDC in the ipsilateral breast.

**Case Report:**

A 48-year-old woman presented with a palpable mass in her right breast. Preoperative dynamic contrast-enhanced magnetic resonance imaging (DCE-MRI) demonstrated an irregularly shaped mass with internal rim enhancement. Surgical resection confirmed IDC of nuclear grade 3 with a high proliferation index (Ki-67: 70%), and the patient underwent adjuvant chemotherapy without radiation. Five months postoperation, a chest computed tomography (CT) revealed a new round-shaped lesion with rim enhancement and relatively circumscribed margins near the previous operation site. Breast ultrasound additionally identified a complex cystic and solid mass with an echogenic rind and increased vascularity. Following total resection, a pure squamous cell carcinoma with prominent keratinization was confirmed.

**Conclusion:**

Accurate and early diagnosis of synchronous multiple primary malignancies from recurrence of the primary tumor is critical for improving prognosis by establishing an appropriate treatment and follow-up plan. Recognizing complex cystic and solid masses with relatively circumscribed margins on radiological imaging can assist clinicians in identifying and managing rare cases where IDC and SCC coexist or appear sequentially within a short period.

## 1 INTRODUCTION

Multiple primary malignancies are rare and are defined as the occurrence of two or more distinct cancers in an individual, classified based on their timing and pathological characteristics [1]. Regarding epidemiological classification, multiple primary malignancies may be present in either a single or multiple anatomical location, with each malignancy showing distinct histological features confirming their independent origin rather than metastasis from a primary site [2]. These malignancies can arise synchronously within 6 months or metachronously over time during follow-up. Rare instances of multiple primary malignancies can be observed in the breast, including the extremely uncommon synchronous occurrence of squamous cell carcinoma (SCC) and invasive ductal carcinoma (IDC), which has been reported in only a few cases [3]. Herein, we present a unique case highlighting this rare coexistence at the primary surgical site. Recent emerging studies have increasingly focused on the imaging differentiation and prognostic assessment of rare breast malignancies, including squamous cell carcinoma (SCC) and metaplastic carcinoma [4]. However, literature regarding the diagnostic radiologic clues and prognostic impact of synchronous primary breast malignancies remains sparse. This case report aims to contribute valuable clinical insights into the early radiological differentiation of synchronous SCC from recurrent invasive ductal carcinoma (IDC), as well as underline the prognostic significance associated with this rare co-occurrence.

## 2 CASE REPORT

**Fig. (1):**
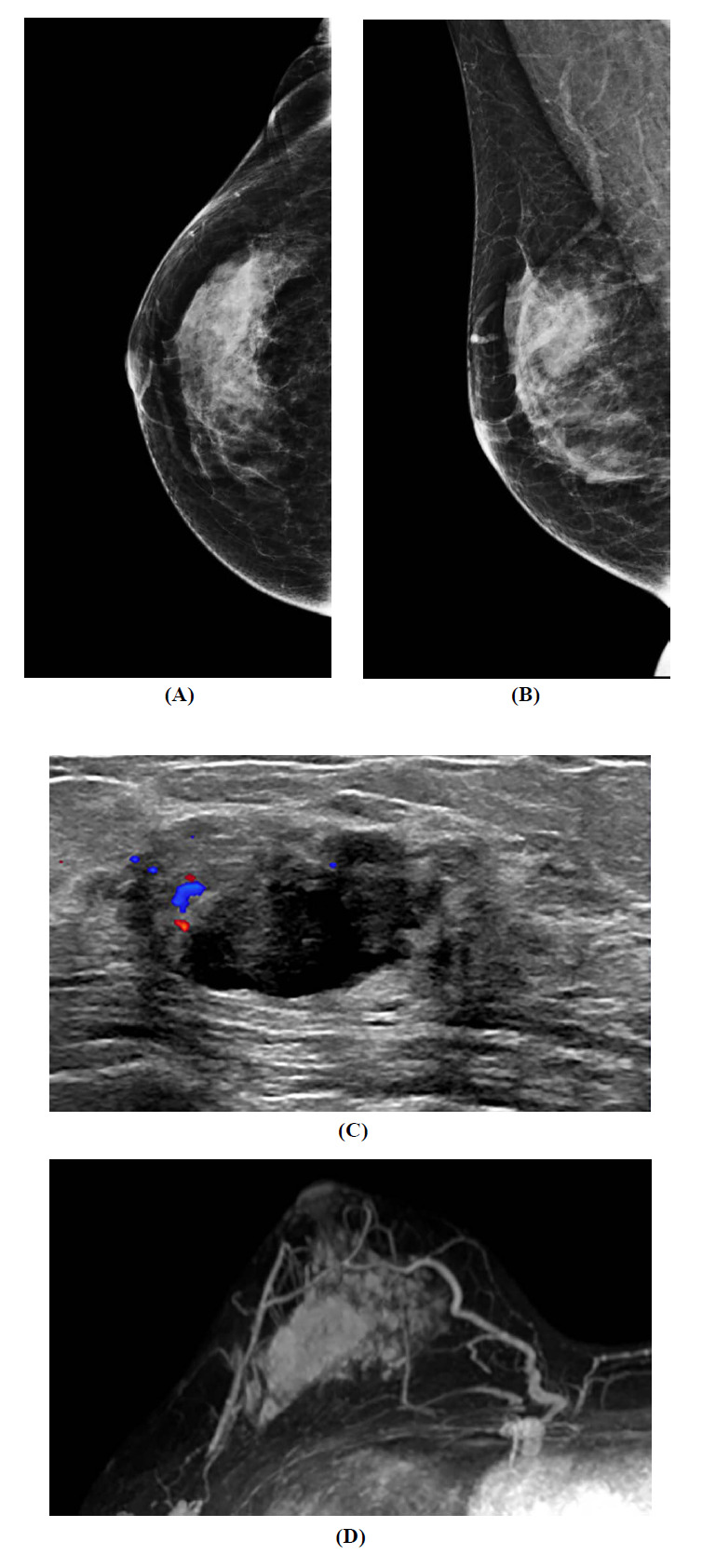
Findings of imaging study of the patient with initial invasive ductal carcinoma of the right breast. (**A**) Craniocaudal and (**B**) mediolateral oblique mammographic views reveal a 2.5 x 2.6 cm iso-dense, irregularly shaped mass with obscured margins located in the upper outer quadrant. (**C**) Ultrasound image shows a 3.2 x 1.5 x 2.8 cm sized irregularly shaped solid mass, with indistinct margins and increased vascularity on Doppler sonography. (**D**) Positivity for the adjacent vessel sign and prominent increased ipsilateral whole-breast vascularity in IDC-confirmed mass were observed on the maximum-intensity-projection image of the breast dynamic contrast-enhanced magnetic resonance imaging.

A 48-year-old premenopausal woman presented with a palpable mass in the right breast, which she noticed for approximately 6 weeks. There was no family history of breast or ovarian cancer; however, her mother had been diagnosed with uterine cancer. The patient had no history of systemic diseases, other primary malignancies, hormone replacement therapy, or oral contraceptive use. A 3 × 3 cm mass was palpable at the 10 o’clock position, approximately 3 cm away from the nipple, in the right breast on physical examination. Initial imaging using breast mammography revealed a 2.5 × 2.6 cm iso-dense irregularly shaped mass and an obscured margin in the upper outer quadrant of the right breast (Fig. **1A** and **B**). A 3.2 × 1.5 × 2.8 cm irregularly shaped solid mass, with indistinct margins and increased vascularity, was seen on color Doppler ultrasound (Fig. **1C**). There was no suspicious lymphadenopathy in the axillary or supraclavicular areas. Ultrasound-guided core needle biopsy confirmed invasive ductal carcinoma (IDC). Immunohistochemical analysis revealed positive staining for estrogen (ER) and progesterone receptors (PR) and negative staining for human epidermal growth factor receptor 2 (HER2). A preoperative dynamic contrast-enhanced magnetic resonance imaging (DCE-MRI) was performed to characterize the extent of the disease. The imaging showed a 3.1 × 1.9 × 3.1 cm mass in the upper outer quadrant of the right breast with an irregular shape, margin, and rim enhancement; however, no suspicious axillary lymph nodes were observed. A positive adjacent vessel sign and markedly increased ipsilateral whole-breast vascularity were noticed on the maximum intensity projection image of the breast (Fig. **1D**). Following confirmation of the absence of metastases in the contralateral breast and axillary regions, the patient underwent right breast-conserving surgery. Histopathology of the excised lesion confirmed no specificity of the IDC. The tumor measured 3.2 × 1.7 cm, corresponding to pathological stage pT2, with a <25% intraductal component. Histopathological examination confirmed the tumor as IDC with nuclear grade 3, consistent with the high proliferation index (Ki-67 of 70%). The resected margins were not malignant and had no lymphatic or venous invasion. Of the nine lymph nodes examined, one sentinel lymph node showed micrometastasis, whereas all three axillary nodes were negative for metastasis, resulting in the pathological staging of pT2N1mi. Immunohistochemistry revealed positive ER and PR and negative for HER2. Post-surgery, the patient received over four cycles of adjuvant chemotherapy comprising adriamycin (96 mg) and cyclophosphamide (960 mg). Adjuvant radiation therapy was not administered after breast-conserving surgery for IDC in this case.

**Fig. (2):**
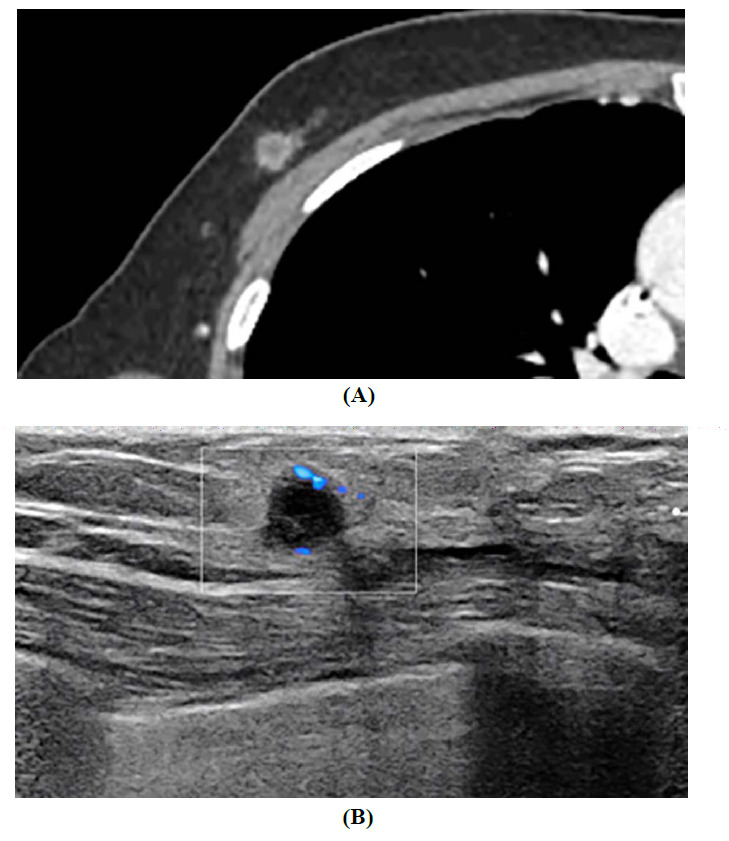
Findings of imaging study of the patient with synchronous primary pure squamous cell carcinoma and invasive ductal carcinoma of the breast. (**A**) Five months post-operation for IDC of the breast. Chest computed tomography showed a 0.9 x 0.9 x 0.7 cm sized rim enhancing mass with a round shape and relatively circumscribed margin near the previous operation site of the right breast. (**B**) Breast ultrasound identified a 0.7 x 0.5 x 0.8 cm complex cystic and solid mass with an echogenic rind at the same site, along with increased vascularity on Doppler sonography.

**Fig. (3):**
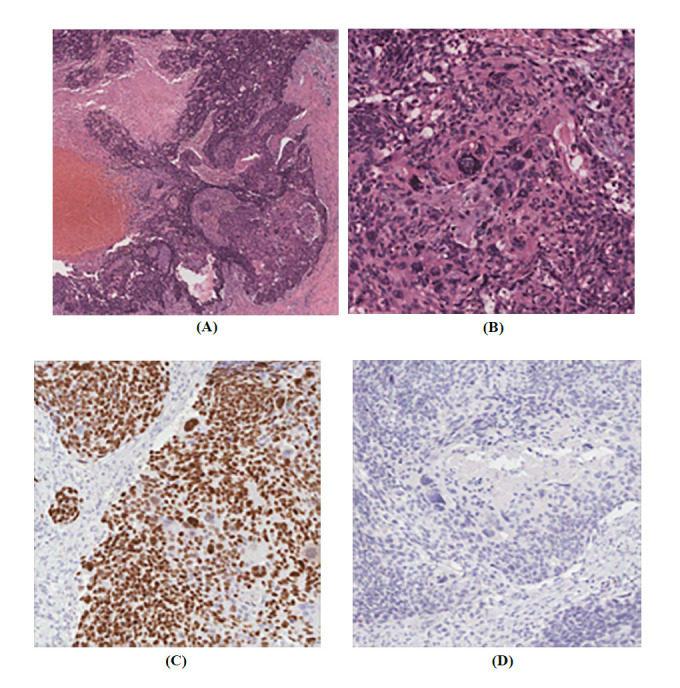
Histopathological findings of the primary pure squamous cell carcinoma of the breast. (**A**) Low-power view showing the overall architecture of the tumor, with nests and sheets of malignant squamous cells invading the breast parenchyma (hematoxylin-eosin [H&E] stain, original magnification: x40). (**B**) High-power view highlighting the prominent keratinization (H&E stain, original magnification: x200). (**C**) p40 immunohistochemistry showing strong nuclear positivity in tumor cells (Original magnification: x200). (**D**) Estrogen receptor immunohistochemistry showing negativity in the tumor cells (Original magnification: x200).

Five months post-operation, chest computed tomography (CT) showed a round-shaped mass of 0.9 × 0.9 × 0.7 cm with an enhanced rim and relatively circumscribed margins near the previous operation site (Fig. **2A**). In addition, breast ultrasound identified a 0.7 × 0.5 × 0.8 cm complex cystic and solid mass with an echogenic rind at the same site, with increased vascularity on Doppler ultrasound (Fig. **2B**). Ultrasound-guided core-needle biopsy of the new lesion revealed an invasive breast carcinoma with squamous differentiation. The pathology report suggested a potential metaplastic carcinoma. Following complete surgical resection, pure squamous cell carcinoma with prominent keratinization was confirmed (Fig. **3A** and **B**). There was no evidence of glandular differentiation or lymphatic or vascular invasion. The tumor was non-adherent to the skin, and the resected margins were tumor-free. Microscopy revealed positive p40, indicating squamous cell differentiation (Fig. **3C**). Immunohistochemistry confirmed a triple-negative status, with negative ER, PR, and HER2 expression and a Ki-67 proliferation index of 60% (Fig. **3D**). Subsequent comprehensive chest, abdominopelvic, and positron emission tomography CT revealed no evidence of primary SCC outside the breast. Subsequently, the patient received curative radiation with volumetric arc therapy and intensity-modulated radiotherapy to the tumor bed at 50 Gy in 20 fractions and to the whole right breast at 45 Gy in 20 fractions. However, 17 months after the second complete surgical resection, the patient developed multiple metastases to the right axilla and lungs.

## 3 DISCUSSION

Synchronous tumors, defined as multiple primary tumors diagnosed within 6 months, are rare clinical manifestations [5]. Generally, these tumors have a poorer prognosis than single breast cancers, even when both are at the same stage [6]. Synchronous and metachronous bilateral breast cancers have a worse prognosis than unilateral breast cancer; however, they exhibit better survival rates than recurrent breast cancers. Furthermore, multiple primary malignancies can have a more favorable prognosis than recurrence. This distinction may arise due to the early detection of multiple primary malignancies and consideration as independent tumors, allowing for possible treatment optimization for each malignancy. In addition, the biological characteristics of multiple primary malignancies may be less aggressive, which may contribute to comparatively better outcomes [7]. Supporting this, a study comparing ipsilateral breast cancer recurrence and multiple primary breast cancers in patients with early breast cancer found that patients with ipsilateral breast cancer recurrence had lower survival rates than those with multiple primary breast cancers at the same TNM stage [8]. These findings emphasize the critical importance of distinguishing between recurring and multiple primary malignancies when evaluating prognosis and formulating treatment strategies. Accurate differentiation achieved through imaging and clinicopathological assessments can significantly impact therapeutic decision-making and follow-up protocols.

Primary pure SCC of the breast is extremely rare, accounting for <0.04% of all breast malignancies [9]. Studies have indicated that primary pure SCC progresses rapidly with a poorer prognosis than IDC [10]. This is due to its distinct biological and clinical characteristics, which are deeply rooted in its epithelial cell origins. SCC arises from the metaplastic transformation of glandular epithelial cells into squamous cells, often exhibiting a basal-like phenotype, characterized by ER-, PR-, and HER2-negativity, and CK5/6-positivity, with high proliferative activity and resistance to targeted therapies like hormone and HER2-directed treatments [11]. Moreover, SCC often presents in advanced stages with larger tumor sizes and greater local invasiveness than IDC. Its epithelial origin and associated mutations, including those in tumor protein p53 and phosphatidylinositol-4,5-biphosphate 3-kinase catalytic subunit alpha, activate aggressive molecular pathways, such as Wnt and mechanistic target of rapamycin, further enhancing adaptability and progression [10]. In addition, SCC has demonstrated low responses to chemotherapy and limited benefits from radiotherapy, complicating its management [11]. These factors collectively result in a poorer prognosis for SCC compared to IDC, highlighting the challenges in treating this rare and aggressive subtype. Although previous studies reported cases of radiation-induced secondary breast malignancies, including SCC [12], this case notable developed SCC without prior radiation therapy, indicating that other intrinsic biological factors, such as high nuclear grade and Ki-67 proliferation index, may have contributed significantly to the metaplastic transformation and aggressive progression.

Distinguishing primary pure SCC of the breast from recurrent IDC before surgery is challenging owing to the lack of specific radiological findings; however, it is essential to tailor treatment strategies and improve prognosis. Fine-needle aspiration and core needle biopsy are commonly used for breast cancer diagnosis; nevertheless, they may not capture all tumor characteristics owing to limited tissue sampling. Primary SCC often includes an IDC component. Solely relying on the IDC portion obtained through fine-needle aspiration or core needle biopsy can lead to diagnostic errors and affect clinical judgment. Imaging can play a complementary role in diagnosis. IDC typically presents as an irregular mass on tomosynthesis and ultrasound and as an enhancing mass with an irregular shape and margins on MRI [13]. In contrast, SCC lacks well-defined radiologic characteristics; however, it often shows cystic characteristics, as noted in this case [14]. Metaplastic breast carcinoma with squamous cell differentiation typically presents as a mildly lobulated mass with mixed echogenicity on ultrasound imaging, showing solid and cystic components. These cystic regions correlated with the necrosis and cystic degeneration observed in histopathology [15]. The mammographic features of metaplastic carcinoma, including SCC, often mimic benign characteristics, such as an oval shape and circumscribed margins [4]. Breast MRI can aid in differentiating challenging cases with greater sensitivity and specificity. SCC and IDC often appear as round to lobular masses with smooth margins and similar signal intensities on T1-weighted images. However, SCC frequently shows high signal intensity on T2-weighted images due to necrosis or cystic degeneration, a feature shared by other breast cancer subtypes. Furthermore, SCC commonly exhibits heterogeneous or rim-like enhancement patterns with early enhancement and delayed washout, similar to our case with rim-like enhancement on MRI [15]. Therefore, multimodal imaging could help the clinician distinguish primary pure SCC of the breast from recurrent IDC following complete surgical resection. However, future studies could investigate these imaging features in a larger cohort to validate their diagnostic accuracy and clinical utility.

## CONCLUSION

In conclusion, accurate differentiation of synchronous multiple primary malignancies from recurrent breast carcinoma is essential for optimizing prognosis through appropriate treatment and follow-up. This case underscores the importance of recognizing specific imaging characteristics, such as complex cystic and solid features with rim enhancement, which may serve as critical diagnostic clues for clinicians managing rare cases of synchronous IDC and SCC.

## Data Availability

The data that support the findings of this study are available within the article.

## LIST OF ABBREVIATIONS

SCC: Squamous cell carcinoma
IDC: Invasive ductal carcinoma
ER: Estrogen receptor
PR: Progesterone receptor
HER2: Human epidermal growth factor receptor 2
CT: Computed tomography
DCE-MRI: Dynamic contrast-enhanced magnetic resonance imaging
H&E: Hematoxylin and eosin

## STANDARDS OF REPORTING

CARE guidelines were followed.
